# Comparative transcriptome and metabolome analyses of four *Panax* species explore the dynamics of metabolite biosynthesis

**DOI:** 10.1016/j.jgr.2022.07.001

**Published:** 2022-07-16

**Authors:** Hyunjin Koo, Yun Sun Lee, Van Binh Nguyen, Vo Ngoc Linh Giang, Hyun Jo Koo, Hyun-Seung Park, Padmanaban Mohanan, Young Hun Song, Byeol Ryu, Kyo Bin Kang, Sang Hyun Sung, Tae-Jin Yang

**Affiliations:** a Department of Agriculture, Forestry and Bioresources, Plant Genomics and Breeding Institute, College of Agriculture and Life Sciences, Seoul National University, Seoul, Republic of Korea; b Department of Agricultural Biotechnology, Plant Genomics and Breeding Institute, College of Agriculture and Life Sciences, Seoul National University, Seoul, Republic of Korea; c College of Pharmacy and Research Institute of Pharmaceutical Sciences, Seoul National University, Seoul, Republic of Korea; d Research Institute of Pharmaceutical Sciences, College of Pharmacy, Sookmyung Women's University, Seoul, Republic of Korea

**Keywords:** Adventitious root, *De novo* assembly, Oxidosqualene cyclase, *Panax* species, Specialized metabolite profiling

## Abstract

**Background:**

The genus *Panax* in the Araliaceae family has been used as traditional medicinal plants worldwide and is known to biosynthesize ginsenosides and phytosterols. However, genetic variation between *Panax* species has influenced their biosynthetic pathways is not fully understood.

**Methods:**

Simultaneous analysis of transcriptomes and metabolomes obtained from adventitious roots of two tetraploid species (*Panax ginseng* and *P. quinquefolius*) and two diploid species (*P. notoginseng* and *P. vietnamensis*) revealed the diversity of their metabolites and related gene expression profiles.

**Results:**

The transcriptome analysis showed that *2,3-OXIDOSQUALENE CYCLASEs* (*OSC*s) involved in phytosterol biosynthesis are upregulated in the diploid species, while the expression of *OSC*s contributing to ginsenoside biosynthesis is higher in the tetraploid species. In agreement with these results, the contents of dammarenediol-type ginsenosides were higher in the tetraploid species relative to the diploid species.

**Conclusion:**

These results suggest that a whole-genome duplication event has influenced the triterpene biosynthesis pathway in tetraploid *Panax* species during their evolution or ecological adaptation. This study provides a basis for further efforts to explore the genetic variation of the *Panax* genus.

## Graphical abstract

**Figure:**
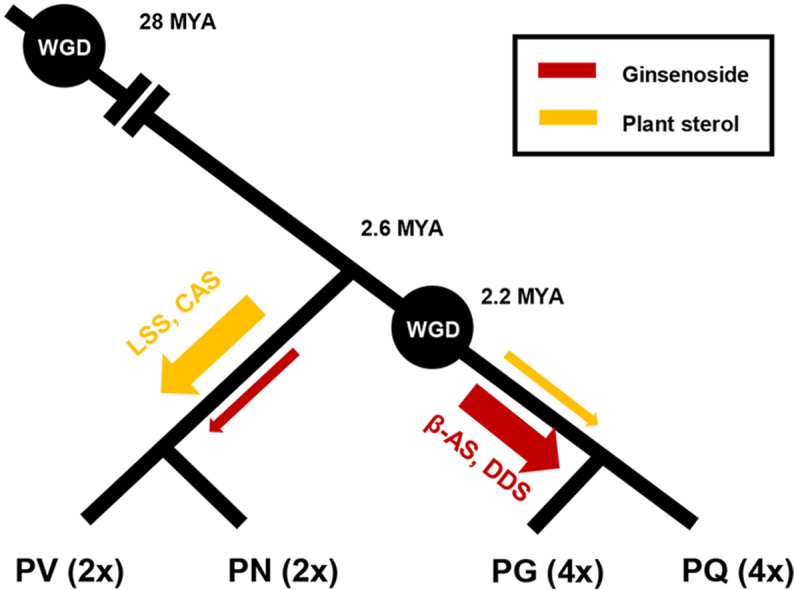

## 1 Introduction

**Fig. 1:**
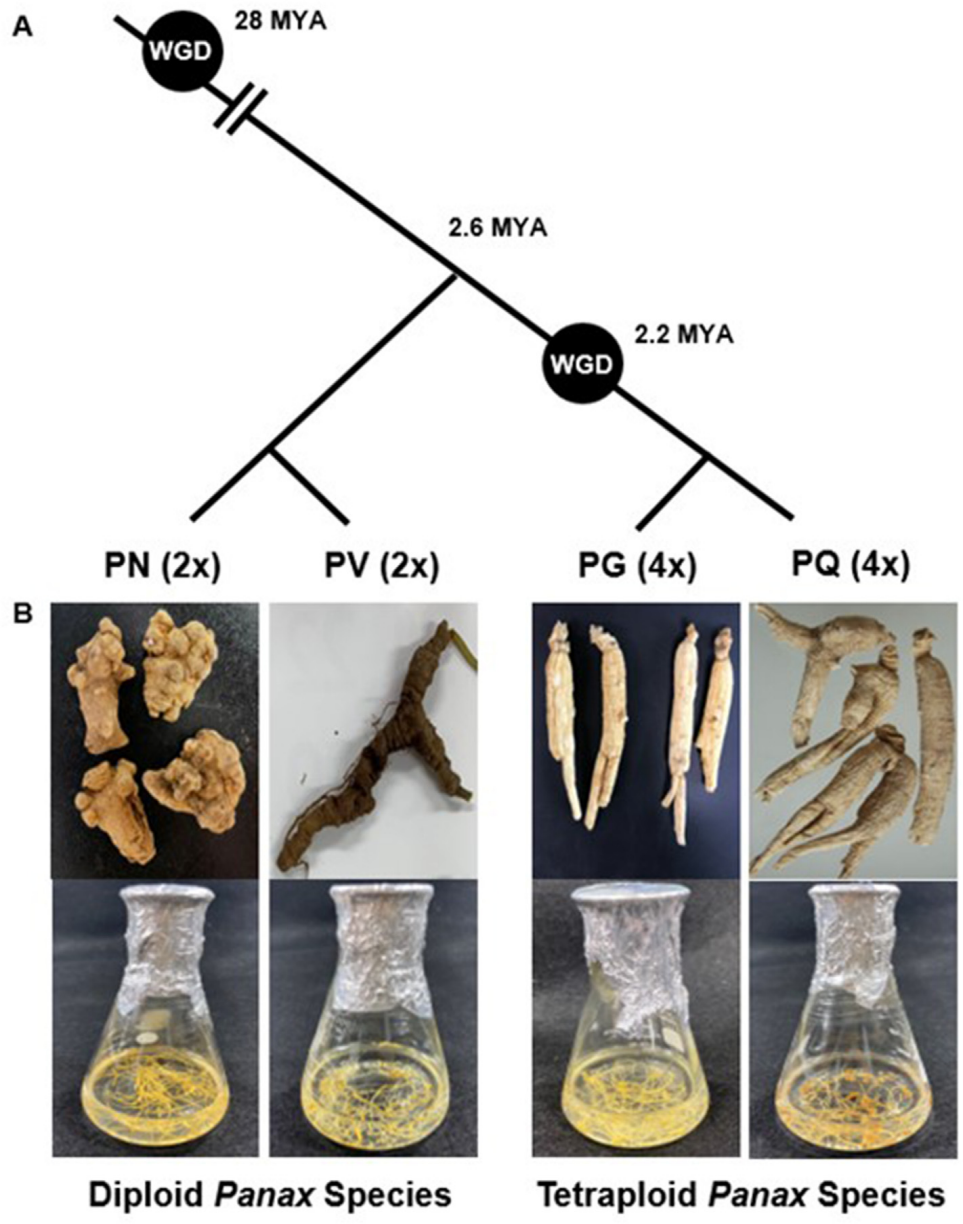
**Phylogenetic relationship and plant materials of four *Panax* species**. (A) Schematic diagram showing the relationships among four *Panax* species. Whole genome duplication (WGD) events are approximately estimated based on previous study [3,4]. **(B)** The adventitious roots maintained in the flasks were collected for transcriptiome and metabolites analysis. PG, *P. ginseng*; PQ, *P. quinquefolius*; PN, *P. notoginseng*; PV, *P. vietnamensis*, MYA, Million years ago.

Ginseng, a member of the Araliaceae family, is an important medicinal plant, as it accumulates many highly valued bioactive compounds [1]. The *Panax* genus involves more than 15 species [2], of which *Panax ginseng* (PG, known as Korean ginseng)*, P. quinquefolius* (PQ, American ginseng)*, P. notoginseng* (PN, Sanchi ginseng) and *P. vietnamensis* (PV, Vietnamese ginseng) are widely used in Asia and North America. The *Panax* species experienced a shared whole-genome duplication (WGD) event about 28 million years ago (MYA). PN and PV are diploid *Panax* species, growing at high altitudes (over 1,600 meters above sea level) in warm regions from Southern China to Southern Vietnam*.* The tetraploid species PG and PQ underwent one additional WGD event about 2.2 MYA, as their gene content is about twice that of diploid *Panax* species, and grow and overwinter widely in North East Asia and North East America (Fig. 1A) [3,4]. This evolutionary process may have influenced the complement of specialized metabolites that accumulate in *Panax* species.

The major *Panax* species retained the core biosynthetic pathway for ginsenosides, which are triterpene glycosides and the major bioactive components of *Panax* species. The biosynthesis of ginsenosides starts from 2,3-oxidosqualene, which is derived from isopentenyl pyrophosphate (IPP) produced through the mevalonate (MVA) pathway [5,6]. Cyclization of 2,3-oxidosqualene is the committed step of triterpenoid and phytosterol biosynthesis and is catalyzed by oxidosqualene cyclases (OSCs) including dammarenediol-II synthase (DDS), beta-amyrin synthase (β-AS), lanosterol synthase (LSS), cycloartenol synthase (CAS) and lupeol synthase (LUS) [[7], [8], [9]]. In particular, dammarane-type ginsenosides are abundant in the four *Panax* species [[10], [11], [12], [13]]. The biosynthesis of dammarenediol-II is mediated by DDS, followed by its hydroxylation by protopanaxadiol synthase (PPDS) to generate protopanaxadiol (PPD). Subsequently, PPD is itself hydroxylated by protopanaxatriol synthase (PPTS) to produce protopanaxatriol (PPT). Various dammarane-type ginsenosides are also produced by glycosylation of the PPD and PPT backbone.

Each *Panax* species biosynthesizes its characteristic ginsenosides. Liquid chromatography–mass spectrometry (LC–MS)-based untargeted metabolomics studies revealed the extensive chemical diversity of *Panax* species [14,15]. Several studies identified the putative genes involved in ginsenoside biosynthesis in PG [14,16,17], PQ [16,18], PN [18,19] and PV [20]. However, how genetic variation between *Panax* species has influenced their biosynthetic pathways of triterpenoids and phytosterols has not been studied in detail due to the lack of an integrated analysis of transcriptome and metabolome data.

In this study, we performed transcriptome and metabolome analyses of adventitious roots collected from PQ, PG, PN, and PV grown under controlled environmental conditions. We compared the transcript levels of candidate genes encoding the proteins involved in the triterpenoid and phytosterol biosynthesis pathways, from the branch point of the committed step in the two pathways to the major types of ginsenosides. Additionally, we analyzed the difference in the composition and abundance of ginsenosides in each species. These findings reveal a coincidence between the ploidy levels and the metabolic profiles for ginsenoside and phytosterol across *Panax* species, suggesting that WGDs have exerted an important influence on the metabolic diversity in *Panax* species.

## 2 Materials and methods

### 2.1 Plant materials

Adventitious roots of *Panax ginseng, P. quinquefolius, P. notoginseng* and *P. vietnamensis* were obtained by growth under previously described conditions [21]. The adventitious roots from the same cultivars were divided, transferred into three bioreactors as independent biological replicates, and collected 4 weeks later for RNA extraction and LC–MS analysis.

### 2.2 RNA extraction and transcriptome deep sequencing (RNA-seq)

Total RNA was extracted using a Plant RNeasy mini kit (Qiagen, Hilden, Germany). The quantity and quality of total RNA were evaluated by agarose gel electrophoresis and on a Nanodrop spectrophotometer (Thermo Fisher Scientific, Waltham, MA, USA). Around 1 μg total RNA was used for the construction of sequencing libraries, which were sequenced on an Illumina NextSeq500 instrument as 150-bp paired-end reads by Lab Genomics (Pankyo, Korea). The raw RNA-seq data for PQ, PN and PV were deposited in the National Center for Biotechnological Information (NCBI) Sequence Read Archive (SRA, http://www.ncbi.nlm.nih.gov/sra) (accession numbers SRR14874116, SRR14874115 and SRR14874114 for PQ; SRR14874113, SRR14874112 and SRR14874111 for PN; SRR14874110, SRR14874109 and SRR14874108 for PV). The raw RNA-seq data from PG were obtained from previous reports (accession numbers: SRR1688723, SRR1688724 and SRR619718) [21,22].

### 2.3 *De novo* transcriptome assembly and functional annotation

Roots from PQ, PN and PV were collected in triplicates and subjected to sequencing, generating 64,553,202 reads for PQ, 70,686,274 reads for PN and 80,932,588 reads for PV. The raw reads for PG (122,075,004 reads) were obtained from previous studies [21,22]. The quality of sequencing data was examined using fastQC prior to further analysis. Low-quality reads and adapter sequences were removed from the raw data based on sequence quality and read length using Trimmomatic-0.33 (TRAILING:20 SLIDINGWINDOW:4:15 MINLEN:75) [23]. After pre-processing, the filtered reads were assembled with Trinity *de novo* assembler based on default parameters [24] (Supplementary Table S1). To reduce gene length bias and other bias during assembly, the best open reading frames were determined using TransDecoder. All transcripts of less than 200 bp in length were eliminated before conducting further analysis. Redundancy reduction was performed by clustering highly similar sequences using CD-Hit with an identity threshold of 0.95 [25].

The assembled transcript sequences (unigenes) were annotated via sequence comparison with known protein databases. The assembled unigenes were annotated by homology search using BLASTX against the Nr (NCBI non-redundant protein database, http://www.ncbi.nlm.nih.gov/), TrEMBL and SwissProt (ftp://ftp.expasy.org/databases/uniprot/current_release/knowledgebase/complete/uniprot_sprot.fasta.gz) databases with minimum e-value threshold of 1e^5^. Gene Ontology (GO) analysis was conducted using Blast2GO to identify the putative function of each unigene. GO terms were assigned to transcripts in three categories (biological process, molecular function and cellular component).

### 2.4 Phlyogenetic analysis with 20 OSCs

Sequence alignments were performed on 20 OSCs protein sequences using MAFFT [3,26]. Phylogenetic tree based on OSCs protein sequence was constructed using maximum likelihood method by performing 1,000 bootstrap replicates [27].

### 2.5 Expression profiling

The filtered reads of each replicate were mapped against the assembled *de novo* transcriptome to quantify transcript abundance. High-quality RNA-seq reads after filtering were aligned to the *de novo* transcriptome using bowtie2 [28]. To estimate unigene abundance, fragments per kilobase exon per million mapped reads (FPKM) values were calculated using RSEM software and filtered to retain unigenes with FPKM > 1 [29]. To compare expression levels across species, candidate transcripts involved in the biosynthesis of bioactive compounds were selected based on the *P. ginseng* reference dataset due to available high-quality genomic resources of the *Panax* species [3]. Orthologous genes from other species were obtained from BLASTX results of transcripts against the *P. ginseng* reference genome (with a minimum identity of 90%, minimum e-value threshold of 1e^5^). Trimmed mean of M-values (TMM) scaling normalization was conducted to normalize differences in total reads across all samples [[30], [31], [32], [33]]. Differentially expressed genes were determined by one-way ANOVA in R (_*P*_ < 0.05) [34].

### 2.6 Untargeted LC–MS profiling of ginsenosides

Ginsenosides profiling was performed as previously described [35]. The list of MS features with peak area was extracted from raw data using MS-DIAL 4.60 [36]. Ginsenosides were putatively annotated by comparing their relative retention time, *m/z* values and major fragment ions of major peaks to reference compounds [[37], [38], [39], [40]]. All raw MS data are publicly available via MassIVE (https://massive.ucsd.edu) under accession number MSV000087966.

## 3 Results

### 3.1 *De novo* transcriptome assembly and functional annotation

**Fig. 2:**
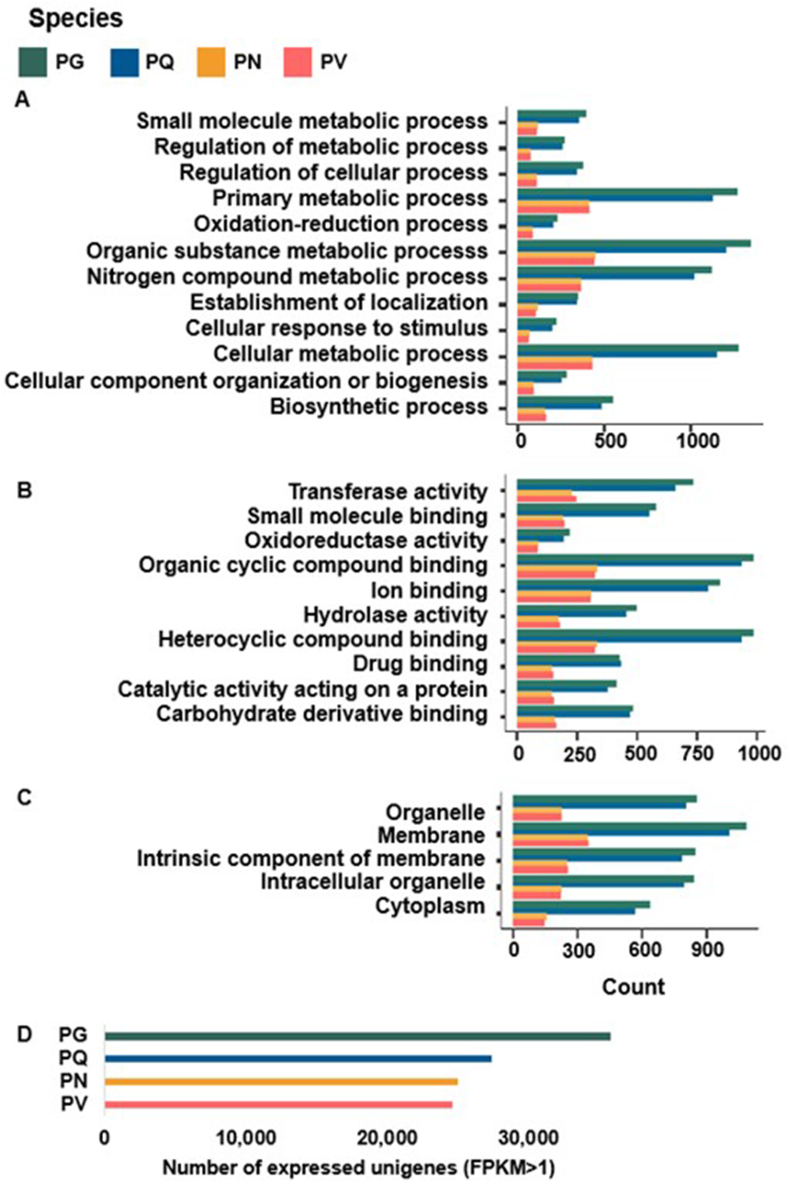
**Gene ontology (GO) annotation of unigenes and transcription factors in the adventitious roots of four *Panax* species**. Counts of gene ontology annotation (level 3) for transcripts in adventitious roots of four *Panax* species has been classified following three distinct aspects of gene functions; **(A)** Biological process, **(B)** Molecular Function and **(C)** Cellular Component. **(D)** Numbers of expressed unigenes (FPKM>1) in adventitious roots of four *Panax* species. PG, *P. ginseng*; PQ, *P. quinquefolius*; PN, *P. notoginseng*; PV, *P. vietnamensis*; FPKM, fragments per kilobase per million.

We analyzed the transcriptome and metabolite profiles of four *Panax* species using adventitious roots: two tetraploid species (PG and PQ) and two diploid species (PN and PV), grown under controlled conditions (Fig. 1). We performed RNA-seq and *de novo* transcriptome assembly to explore the genes responsible for the genetic diversity of the triterpene biosynthetic pathway. We obtained between 290,408 (PQ) and 499,656 (PG) transcripts with an average length ranging from 797 bp (PG) to 1,035 bp (PV) (Table 1). These transcripts defined 60,190 unigenes for PG, 45,309 unigenes for PQ, 43,969 unigenes for PN, and 42,015 unigenes for PV, which we annotated and subjected to GO analysis using Blast2GO (Supplementary Table S2). We then identified the GO categories for all genes within each *Panax* species: 12 biological processes (Fig. 2A), 10 molecular functions (Fig. 2B) and 5 cellular components (Fig. 2C). In the biological process category, GO terms corresponding to ‘organic substance metabolic process’, ‘primary metabolic process’ and ‘cellular metabolic process’ were enriched in the four *Panax* species. The molecular function category consisted of a large proportion of compound binding categories, such as ‘organic cyclic compound binding’ and ‘heterocyclic compound binding’. In the cellular component category, the GO term ‘membrane’ was highly represented.

**Table 1:** ***De novo* Assembly of Unigenes in the Adventitious Roots of Four *Panax* Species.** PG, *P. ginseng*; PQ, *P. quinquefolius*; PN, *P. notoginseng*; PV, *P. vietnamensis.*

Species	Total assembled bases	Median contig length (bp)	Average contig length (bp)	N50	Initial Number of transcripts	Filtered with Transdecoder	Non-redundant transcripts
**PG**	333,067,880	455	796.59	1,070	499,656	165,276	64,514
**PQ**	247,626,301	516	852.68	1,356	290,408	131,713	48,034
**PN**	292,549,562	542	944.42	1,568	309,768	134,212	46,971
**PV**	309,602,171	597	1034.7	1,742	299,219	138,487	44,865

Most GO terms show more than two-fold of unigenes in the tetraploid *Panax* species (PG and PQ) compare to that of the diploid *Panax* species (PN and PV), reflecting maintenance of the most of duplicated genes in the tetraploid *Panax* species [41]. To analyze transcript levels, we mapped the RNA-seq reads to the assembled unigenes and calculated the corresponding FPKM values. After removing genes with low expression (FPKM < 1), we obtained 35,812, 27,451, 25,003 and 24,676 expressed unigenes in PG, PQ, PN and PV, respectively (Fig. 2D) and compared the expression patterns of genes related to the ginsenoside biosynthesis pathway across the four *Panax* species.

### 3.2 Expression levels of serial genes for phytosterol biosynthesis

**Fig. 3:**
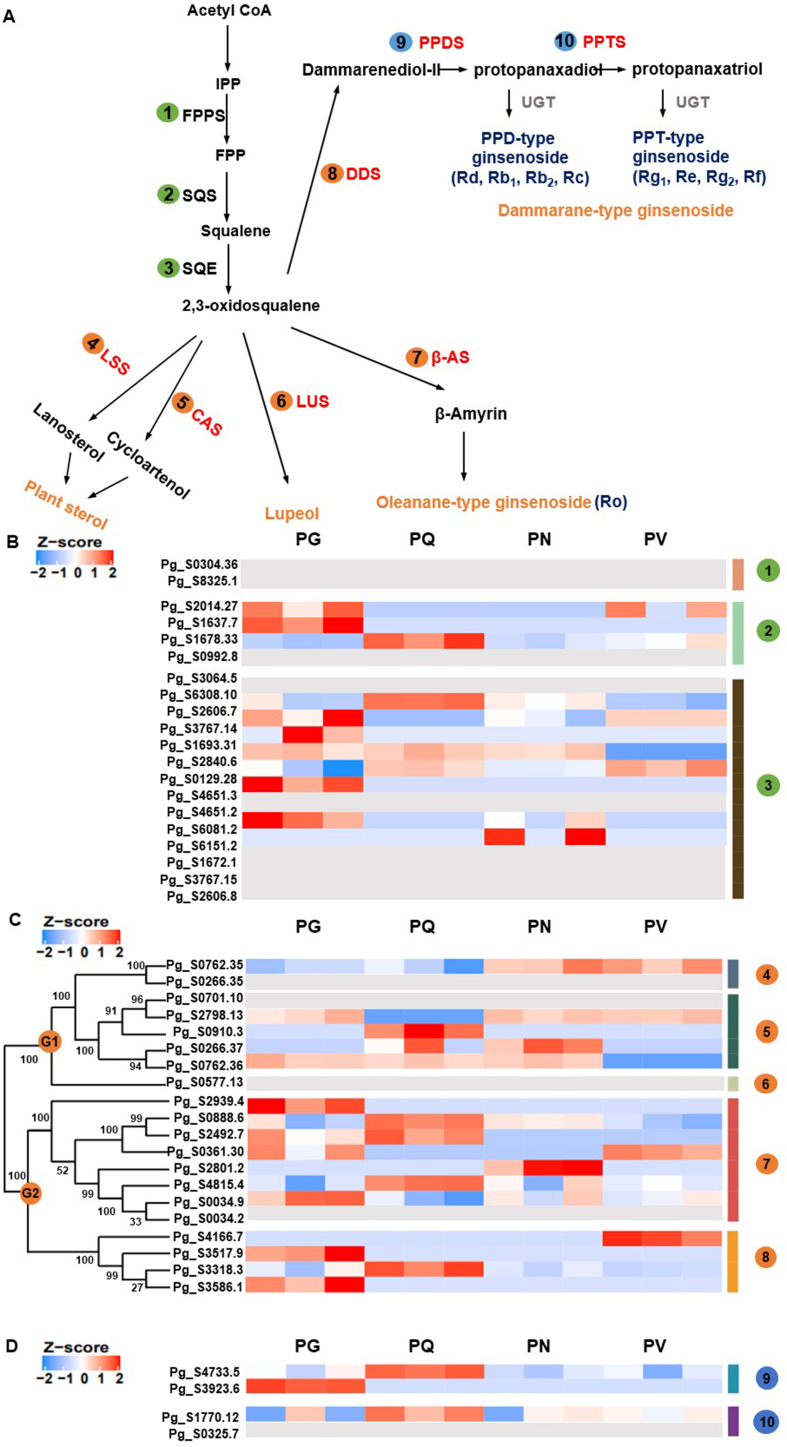
**Putative ginsenoside biosynthetic pathway and expression profiles of the corresponding unigenes in the adventitious roots of four *Panax* species (each species with three biological replicates)**. **(A)** Putative ginsenoside biosynthetic pathway in the *Panax* species; β-AS, β-amyrin synthase; CAS, cycloartenol synthase; DDS, dammarenediol synthase; FPPS, farnesyl diphosphate synthase; IPP, isopentenyl diphosphate; LSS, lanosterol synthase; LUS, lupeol synthase; PPDS, protopanaxadiol synthase; PPTS, protopanaxatriol synthase; SQE, squalene epoxidase; SQS, squalene synthase; UGT, UDP-glycosyltransferase. **(B)** Expression level of transcripts corresponding to FPPS, SQS and SQE. **(C)** Phylogenetic tree based on OSC reference genes and expression level of transcripts corresponding to OSCs in four *Panax* species. Bootstrap values for maximum likelihood analysis are represented on the nodes. **(D)** Expression level of transcripts corresponding to dammarenediol type ginsenoside biosynthesis; OSCs, oxidosqualene cyclases; LSS, CAS and LUS grouped as G1; β-AS and DDS grouped as G2. Colors denote log_2_(x+1) transformed TMM normalized FPKM values and scaled across each row. Gray color indicates the missing expression data; FPKM, fragments per kilobase per million; TMM, trimmed mean of M values; PG, *P. ginseng*; PQ, *P. quinquefolius*; PN, *P. notoginseng*; PV, *P. vietnamensis*.

We had previously predicted 44 genes as being related to triterpenoid and phytosterol biosynthesis [3] (Fig. 3A). Orthologous genes from four *Panax* species were identified BLASTX results of transcripts against these reference genes. Principal component analysis (PCA) with the expression data for these orthologous genes revealed a clear clustering between four *Panax* species (Supplementary Fig. S1). In the present study, we identified one *LUS* to 14 *SQUALENE EPOXIDASE*, (*SQE*) unigenes involved in triterpenoid and phytosterol biosynthesis, several of which showed different expression patterns in the four *Panax* species (Supplementary Table S3). None of the unigenes corresponding to *FARNESYL PYROPHOSPHATE SYNTHASE* (*FPPS*) were expressed in any species. Unigene Pg_S1678.33 for *SQUALENE SYNTHASE* (*SQS*) was highly expressed in PQ, while the unigene Pg_S1637.7 for *SQS* was specifically detected in PG, with no reads in other species. The unigene Pg_S6308.1 for *SQE* was also highly expressed in PQ, whereas the other *SQE* unigene Pg_S2840.6 was only expressed at high levels in PV (Fig. 3B; Supplementary Table S3).

Among the 20 *OSC* genes identified in our previous study of the PG genome, our transcriptome assembly detected expression for 16 *OSC*, 4 *DDS*, 8 *β-AS*, 5 *CAS*, 2 *LSS* and 1 *LUS* unigenes [3]. *DDS* and *β-AS* involved in the triterpenoid biosynthesis were highly expressed in the tetraploid species PG and PQ, whereas *LSS*, which is associated with phytosterol biosynthesis, was strongly expressed in the diploid species PN and PV. The *DDS* unigene Pg_S3318.3 was commonly expressed in all species, with a significantly higher expression level in the tetraploid species PQ (Fig. 3C; Supplementary Table S3). Pg_S3517.9 and Pg_S3586.1, other *DDS* unigenes, were only detected in PG (Fig. 3C; Supplementary Table S3). Among the unigenes for *β-AS*, which is involved in the biosynthesis of oleanane-type ginsenosides, the two unigenes Pg_S2939.4 and Pg_S2492.7 were specifically detected in the tetraploid species PQ and PG. Three other *β-AS* unigenes (Pg_S0888.6, Pg_S4815.4 and Pg_S0034.9) were highly expressed in PQ or PG (Fig. 3C; Supplementary Table S3). Although most *DDS* and *β-AS* unigenes were strongly expressed in the tetraploid species PG and PQ, two of the eight *β-AS* unigenes, Pg_S2801.2 and Pg_S0361.30, were highly expressed uniquely in PN or PV, respectively. By contrast, Pg_S0762.35 (*LSS*) linked to phytosterol biosynthesis was significantly upregulated in the diploid species PN and PV compared to the tetraploid species PG and PQ. To explore the differences in expression for genes involved in the biosynthesis pathway of dammarane-type ginsenosides, we compared the expression levels of unigenes for *PPDS* and *PPTS*. Of the *PPDS* unigenes, Pg_S4733.5 was expressed at a high level in the tetraploid species PQ compared to other species, while Pg_S3293.6 was specifically expressed in the tetraploid species PG. The *PPTS* unigene Pg_S1770.12 showed the highest expression level in PQ compared to the other species (Fig. 3D; Supplementary Table S3).

### 3.3 Ginsenosides profiling in adventitious roots of four *Panax* species

**Fig. 4:**
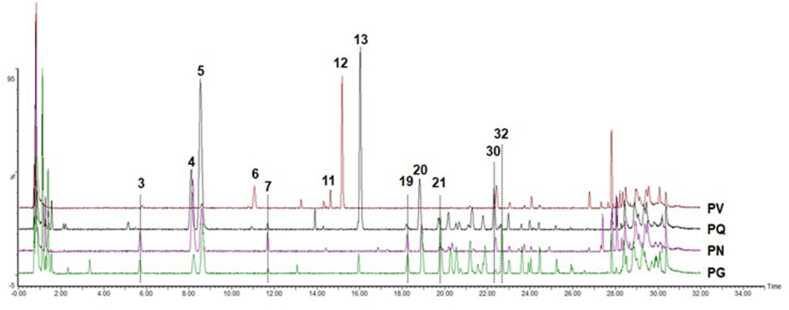
**Representative LC–MS base peak ion chromatograms of the adventitious roots of four *Panax* species (PG, PQ, PN and PV)**. Major chromatographic peaks are denoted with the peak numbers annotated in Supplementary Table S4. PG, *P. ginseng*; PN, *P. notoginseng*; PQ, *P. quinquefolius*; PV, *P. vietnamensis*.

In parallel to RNA-seq, we collected adventitious roots of the four *Panax* species for metabolite profiling by LC–MS (Fig. 4). Among 610 MS features extracted from the data, 48 features were putatively annotated as ginsenosides by the Metabolomics Standards Initiative [42], using identification levels 2 or 3 (Supplementary Table S4). We classified these annotated MS features into PPD-, PPT-, oleanane- or ocotillol-type ginsenosides based on their putative identification.

**Fig. 5:**
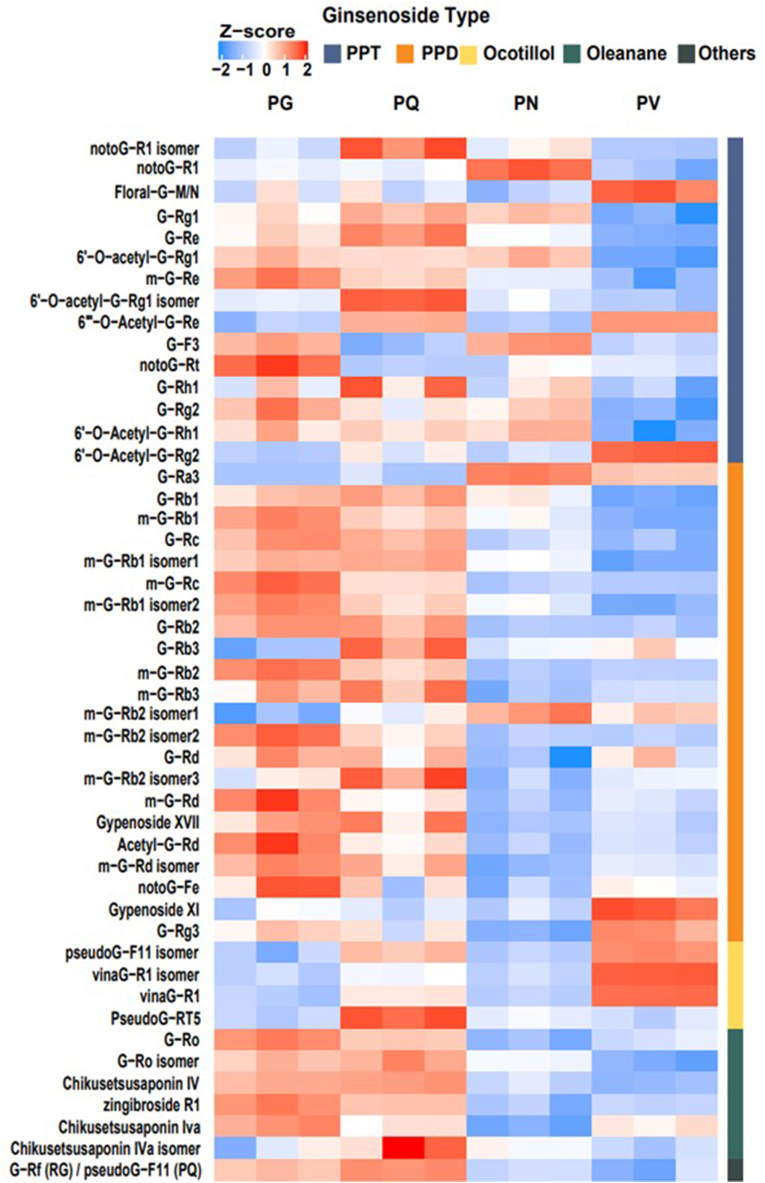
**Relative abundances of putatively identified ginsenosides in the adventitious roots of four *Panax* species (PG, PQ, PN and PV) analyzed by LC–MS (each species with three biological replicates)**. Relative abundance supporting data are denoted in supplementary Table S5. Colors denote log2(x+1) transformed relative abundances of ginsenosides and scaled across each row. PG, *P. ginseng*; PQ, *P. quinquefolius*; PN, *P. notoginseng*; PV, *P. vietnamensis*; PPD, protopanaxadiol; PPT, protopanaxatriol.

PCA with the relative abundance of putative ginsenosides showed a clear separation between the four species (Supplementary Fig. S2), underscoring the interspecific chemical differences between the species. We annotated 22 compounds as PPD-type ginsenosides, of which 18 compounds were significantly more abundant in either or both tetraploid species (Fig. 5; Supplementary Table S4). Several PPD-type ginsenosides such as Ra3, gypenoside XI, Rb isomer and G-Rg3 were, however, abundant in the PN or PV diploid species (Fig. 5; Supplementary Table S5). In particular, Ra3 and gypenoside XI appeared to accumulate to high levels in PN or PV but were present only in trace amounts in the tetraploid species, indicating that these PPD-type ginsenosides are unique to the diploid ginseng species.

We also annotated 15 metabolites as PPT-type ginsenosides. Of those, G-Re was enriched in the tetraploid species PQ, followed by the other tetraploid species PG and the diploid species PN. The two major PPT-type ginsenosides G-Rg2 and G-Rh1 were highly abundant in the PG or PQ tetraploid species, with one minor m-G-Re isomer also accumulating in the tetraploid species. Most PPD-type and PPT-type ginsenosides were characterized by high contents in the tetraploid species PG and PQ, which is consistent with the upregulation of *DDS* transcript levels in these species.

Moreover, six oleanane-type ginsenosides such as chikusetsusaponin-type and Ro significantly accumulated in the PG or PQ tetraploid species, which correlated with the high expression of *β-AS* unigenes (Fig. 5; Fig. 3C). We detected four ocotillol-type ginsenosides in all four species, and the isomer of pseudoginsenoside F11 and vinaG-R1 belonging to ocotillol-type ginsenoside were significantly discovered in the tetraploid PQ and the diploid PV (Fig. 5).

## 4 Discussion

### 4.1 Functional classification of *OSC* genes

Medicinal plants produce a bounty of bioactive compounds such as triterpenoid scaffolds and plant sterols [43,44]. Plant sterols have important roles in phytohormone signaling and are critical structural components of the cell membrane [45]. Triterpenoids are not essential for growth but have commercial applications in the pharmaceutical industry [46,47].

The first committed step in the biosynthesis of plant sterols and triterpenoids is the cyclization of 2,3-oxidosqualene, which is catalyzed by OSCs [48]. OSCs are classified into two groups according to their functions: OSCs associated with plant sterol and lupeol biosynthesis (LSS, CAS and LUS), and OSCs related to dammarane-type and oleanane-type ginsenoside biosynthesis (DDS, β-AS). In our phylogenetic analysis, PG OSCs grouped well based on their predicted functions (Fig. 3), suggesting that their enzymatic functions might be conserved in PG. We aimed to determine if the expression levels of *OSC*s contributed to the accumulation of triterpenoids in different *Panax* species. As specialized metabolism is readily affected by the environment (Fig. 1) [49], we used adventitious roots grown under identical controlled conditions and investigated the transcript levels of the five types of *OSC* genes *(DDS, β-AS, CAS, LSS* and *LUS*) and the contents of ginsenosides in four *Panax* species.

### 4.2 Ploidy level and expression patterns of *OSC* genes

Our integrated analysis revealed a notable pattern in the transcript levels of some *OSC*-related genes, along with their correlation with ploidy levels among *Panax* species (Fig. 3A; Supplementary Table S3) [9]. Autopolyploidization results in an alteration of metabolite contents, gene expression profiles, epigenetic regulation, and protein levels [50]. The influence of polyploidization on specialized metabolism has been also reported in several medicinal plants [51]. In many cases, artificially induced polyploids and natural polyploids produced higher concentrations of specialized metabolites than their diploid counterparts [52]. An example of higher triterpene content was reported in a colchicine-induced tetraploid Indian pennywort (*Centella asiatica*), whose total triterpene content was enhanced by the forced genome doubling [53]. However, polyploidization does not always positively affect specialized metabolism [54]. In our study, while the expression of *OSC*s related to ginsenoside biosynthesis were up- regulated in tetraploid species than diploid species, the expression of *OSC*s related to phytosterol biosynthesis were opponent. The naturally occurring genome duplication in the *Panax* species might have exerted an influence towards ginsenoside biosynthesis by affecting gene copy number and the expression of *OSC*s*,* which encode the enzyme at the key branching point of the phytosterol and ginsenoside biosynthetic pathways.

Among the multicopy genes, most *DDS* and *β-AS* unigenes were strongly expressed in the tetraploid species PG and PQ compared to the diploid species PN and PV, suggesting that triterpenoid biosynthesis is intensified by WGD in the *Panax* species. Unlike dammarane- and oleanane-type ginsenoside biosynthesis, the biosynthetic pathway of ocotillol-type ginsenosides is largely unknown, which prevents identification of the responsible genes. Ocotillol-type ginsenosides accumulate to high levels in PQ (tetraploid) and PV (diploid) [[55], [56], [57]]. We identified four ocotillol-type ginsenosides with high contents in PQ or PV, which was consistent with the previous study [58]. As the ocotillol-type ginsenoside pathway is not clearly identified, we did not focus on the genes responsible for the biosynthesis of ocotillol-type ginsenosides in this study. However, because high amounts of ocotillol-type ginsenoside were reported in PQ and PV, we hypothesize that the genes related to ocotillol-type ginsenoside biosynthesis evolved independently of the polyploidization event, unlike the genes associated with the biosynthesis of other types of ginsenosides. Several genes were significantly upregulated in PQ and PV and might constitute good candidate genes for the biosynthesis of ocotillol-type ginsenosides. Further dissection of the PQ and PV genomes may help uncover the genes responsible for ocotillol-type ginsenoside biosynthesis.

### 4.3 Habitats and phytosterol biosynthesis

A possible reason to explain the advantages linked to *Panax* species of different ploidy levels that lead to gene expression bias may call upon their natural growth environments. The *Panax* diploid species PN and PV naturally grow in the warm climate of Southern Asia without winter, whereas the *Panax* tetraploid species PG and PQ overwinter in Northeast Asia and North America [3]. Total phytosterol content is consistently higher with increasing temperatures in soybean (*Glycine max*) seeds [59]. Therefore, we can infer that the high expression of *OSC*s involved in plant sterol biosynthesis in the diploid *Panax* species might be due to their ecological adaptation to warm climate environments. By contrast, the finding that *OSC*s leading to ginsenoside biosynthesis are highly expressed in tetraploid *Panax* species may be related to their growing environments to overcome freezing during the winter season. Duplicated genes might enhance the adaptability to severe environments of polyploid species by increasing global gene expression [60].

In conclusion, our data indicate that the accumulation pattern of ginsenosides correlates with the expression level of various *OSC* genes and with polyploidy level in *Panax* species, suggesting that dammarane-type and oleanane-type ginsenoside biosynthesis pathways are upregulated in tetraploid *Panax* species, likely due to the genome duplication event.
