# Supplementary material for: Comparative transcriptome and metabolome analyses of four Panax species explore the dynamics of metabolite biosynthesis
Source: J Ginseng Res. 2022 Jul 16;47(1):44–53. doi: 10.1016/j.jgr.2022.07.001 (PMC9834023; doi:10.1016/j.jgr.2022.07.001)
Supplement: Multimedia component 1 [file mmc1.docx]

|  |  | **Raw** | | **After filtering** | | | | |
| --- | --- | --- | --- | --- | --- | --- | --- | --- |
| **Species** | **Sample Name** | **Total reads** | **Length (bp)** | **Both Surviving Read** | **Length (bp)** | **Forward Only Surviving Read** | **Reverse Only Surviving Read** | **Drop** |
| **PG^*^** | SRR1688723.1 | 14,306,820 | 1,977,938,763 | 11,937,186 (83.44%) | 1,621,032,382 | 1,228,706 (8.59%) | 410,398 (2.87%) | 730,530 (5.11%) |
|  | SRR1688724.1 | 17,526,160 | 2,446,652,196 | 14,587,380 (83.23%) | 2,001,847,596 | 1,639,918 (9.36%) | 459,646 (2.62%) | 839,216 (4.79%) |
|  | SRR619718 | 90,242,024 | 9,114,444,424 | 65,199,696 (72.24%) | 6,478,560,844 | 2,873,860 (3.18%) | 6,502,262 (7.2%) | 432,182 (0.48%) |
| **PQ** | SRR14874116 | 21,243,678 | 3,167,086,520 | 16,751,646 (78.85%) | 2,433,152,690 | 3,464,782 (16.31%) | 334,728 (1.58%) | 692,522 (3.26%) |
|  | SRR14874115 | 22,510,750 | 3,278,296,935 | 12,675,016 (56.31%) | 1,783,675,070 | 8,321,114 (36.97%) | 323,658 (1.44%) | 1,190,962 (5.29%) |
|  | SRR14874114 | 20,798,774 | 3,080,552,486 | 15,679,874 (75.39%) | 2,257,607,007 | 4,055,418 (19.50%) | 308,118 (1.48%) | 755,364 (3.63%) |
| **PN** | SRR14874113 | 23,721,250 | 3,519,218,114 | 19,031,598 (80.23%) | 2,774,888,793 | 3,423,742 (14.43%) | 426,674 (1.80%) | 839,236 (3.54%) |
|  | SRR14874112 | 25,254,434 | 3,632,381,754 | 17,516,284 (69.36%) | 2,473,959,938 | 5,901,708 (23.37%) | 449,140 (1.78%) | 1,387,302 (5.49%) |
|  | SRR14874111 | 21,710,590 | 3,185,167,373 | 16,639,748 (76.64%) | 2,413,239,980 | 3,579,686 (16.49%) | 472,256 (2.18%) | 1,018,900 (4.69%) |
| **PV** | SRR14874110 | 25,686,128 | 3,772,004,809 | 20,852,030 (81.18%) | 3,025,240,331 | 3,155,246 (12.28%) | 556,616 (2.17%) | 1,122,236 (4.37%) |
|  | SRR14874109 | 26,490,620 | 3,833,224,506 | 20,373,932 (76.91%) | 2,919,027,599 | 4,228,320 (15.96%) | 544,334 (2.05%) | 1,344,034 (5.07%) |
|  | SRR14874108 | 28,755,840 | 4,238,505,638 | 22,923,398 (79.72%) | 3,337,377,227 | 3,866,994 (13.45%) | 652,504 (2.27%) | 1,312,944 (4.57%) |

**Supplementary Table S1.** **Summary of transcriptome data in the adventitious roots of four *Panax* species (PG, PQ, PN and PV).** PG, *P. ginseng*; PQ, *P. quinquefolius*; PN, *P. notoginseng*; PV, *P. vietnamensis*.

* The RNA seq data are from the previous studies (Jayakodi et al. 2014; Lee et al. 2017).

**Supplementary Table S2.** **Functional annotation of all unigenes in the adventitious roots of four *Panax* species (PG, PQ, PN and PV).** BLASTX based similarity search was conducted against the protein database (Non-redundant (Nr), Swiss-Prot and TrEMBL) with a cut-off *E*-value of 10^−5.^ PG, *P. ginseng*; PQ, *P. quinquefolius*; PN, *P. notoginseng*; PV, *P. vietnamensis*.

| **DB** | **Species** | **Assigned Transcripts** |
| --- | --- | --- |
| **Nr** | **PG** | 60,138 |
|  | **PQ** | 45,276 |
|  | **PN** | 43,927 |
|  | **PV** | 41,981 |
| **TrEMBL** | **PG** | 59,933 |
|  | **PQ** | 45,134 |
|  | **PN** | 43,720 |
|  | **PV** | 41,837 |
| **Swiss-prot** | **PG** | 44,760 |
|  | **PQ** | 33,851 |
|  | **PN** | 32,711 |
|  | **PV** | 31,197 |
| **Nr +** **Swiss-Prot + TrEMBL** | **PG** | 60,190 |
|  | **PQ** | 45,309 |
|  | **PN** | 43,969 |
|  | **PV** | 42,015 |

**Supplementary Table S3.** **TMM normalized FPKM values of transcripts assigned to FPPS, SQS, SQE, OSCs, PPDS, PPTS and bHLH in the adventitious roots of four *Panax* species (PG, PQ, PN and PV).** The orthologs were isolated from three *Panax* species with highest sequence similarity to the genes in *P. ginseng*. The transcript levels of the orthologs were compared between four *Panax* species. Shadows indicate that the transcript was not expressed (FPKM <1). PG, *P. ginseng*; PQ, *P. quinquefolius*; PN, *P. notoginseng*; PV, *P. vietnamensis*; FPPS, farnesyl diphosphate synthase; SQS, squalene synthase; SQE, squalene epoxidase; OSCs, oxidosqualene cylcases; PPDS, protopanaxadiol synthase; PPTS, protopanaxatriol synthase.

| **GENE** | **Ref** | **PG** | **PQ** | **PN** | **PV** |
| --- | --- | --- | --- | --- | --- |
| **FPPS** | **Pg_S0304.36** | 0 | 0 | 0 | 0 |
|  | **Pg_S8325.1** | 0 | 0 | 0 | 0 |
|  | **Count** | 0 | 0 | 0 | 0 |
| **SQS** | **Pg_S2014.27** | 7.19±2.76 | 0 | 0 | 5.28±3.70 |
|  | **Pg_S1637.7** | 30.56±7.36 | 0 | 0 | 0 |
|  | **Pg_S1678.33** | 6.21±3.24^c^ | 59.91±8.37^a^ | 13.73±4.20^c^ | 27.71±6.24^b^ |
|  | **Pg_S0992.8** | 0 | 0 | 0 | 0 |
|  | **Count** | 3 | 1 | 1 | 2 |
| **SQE** | **Pg_S3064.5** | 0 | 0 | 0 | 0 |
|  | **Pg_S6308.10** | 13.24±15.21^bc^ | 227.27±23.11^a^ | 26.39±5.25^b^ | 3.18±1.42^c^ |
|  | **Pg_S2606.7** | 9.88±9.85^a^ | 0^c^ | 1.10±1.01^bc^ | 3.51±0.03^ab^ |
|  | **Pg_S3767.14** | 1.18±1.60 | 0 | 0 | 0 |
|  | **Pg_S1693.31** | 8.16±2.11 | 8.74±1.79 | 7.01±1.21 | 0 |
|  | **Pg_S2840.6** | 6.91±8.29^b^ | 28.45±4.44^a^ | 10.73±0.80^ab^ | 45.57±14.38^a^ |
|  | **Pg_S0129.28** | 21.33±17.06 | 0 | 0 | 0 |
|  | **Pg_S4651.3** | 0 | 0 | 0 | 0 |
|  | **Pg_S4651.2** | 3.04±1.66 | 0 | 0.52±0.52 | 0 |
|  | **Pg_S6081.2** | 0 | 0 | 1.08±0.97 | 0 |
|  | **Pg_S6151.2** | 0 | 0 | 0 | 0 |
|  | **Pg_S1672.1** | 0 | 0 | 0 | 0 |
|  | **Pg_S3767.15** | 0 | 0 | 0 | 0 |
|  | **Pg_S2606.8** | 0 | 0 | 0 | 0 |
|  | **Count** | 7 | 3 | 6 | 3 |
| **LSS** | **Pg_S0762.35** | 1.03±0.36^b^ | 1.00±1.00^b^ | 5.25±2.13^a^ | 5.59±1.48^a^ |
|  | **Pg_S0266.35** | 0 | 0 | 0 | 0 |
|  | **Count** | 1 | 1 | 1 | 1 |
| **CAS** | **Pg_S0701.10** | 0 | 0 | 0 | 0 |
|  | **Pg_S2798.13** | 11.84±4.67 | 0 | 12.73±2.16 | 12.80±1.75 |
|  | **Pg_S0910.3** | 0 | 4.39±2.39 | 0 | 0 |
|  | **Pg_S0266.37** | 0 | 3.22±4.65 | 5.63±3.07 | 0 |
|  | **Pg_S0762.36** | 20.55±6.05 | 16.58±3.43 | 18.28±2.65 | 0 |
|  | **Count** | 2 | 3 | 3 | 1 |
| **LUS** | **Pg_S0577.13** | 0 | 0 | 0 | 0 |
|  | **Count** | 0 | 0 | 0 | 0 |
| **β-AS** | **Pg_S2939.4** | 3.08±1.28 | 0 | 0 | 0 |
|  | **Pg_S0888.6** | 1.55±2.05^bc^ | 12.29±1.61^a^ | 3.57±0.22^b^ | 0.60±0.69^c^ |
|  | **Pg_S2492.7** | 2.99±2.27 | 6.43±2.40 | 0 | 0 |
|  | **Pg_S2801.2** | 0 | 0 | 3.20±1.79 | 0 |
|  | **Pg_S0361.30** | 2.82±1.84 | 0 | 0 | 3.48±0.77 |
|  | **Pg_S4815.4** | 1.32±1.14^b^ | 12.35±1.72^a^ | 3.04±2.54^b^ | 2.46±0.63^b^ |
|  | **Pg_S0034.9** | 14.26±6.60^a^ | 1.16±1.59^b^ | 4.57±2.65^b^ | 3.42±1.28^b^ |
|  | **Pg_S0034.2** | 0 | 0 | 0 | 0 |
|  | **Count** | 6 | 4 | 4 | 4 |
| **DDS** | **Pg_S4166.7** | 0 | 0 | 0 | 5.63±1.68 |
|  | **Pg_S3517.9** | 29.37±38.00 | 0 | 0 | 0 |
|  | **Pg_S3318.3** | 21.66±16.32^b^ | 290.07±108.18^a^ | 16.05±5.05^b^ | 12.00±1.31^b^ |
|  | **Pg_S3586.1** | 16.97±21.38 | 0 | 0 | 0 |
|  | **Count** | 3 | 1 | 1 | 2 |
| **PPDS** | **Pg_S4733.5** | 21.40±8.31^b^ | 103.28±10.79^a^ | 13.51±3.75^b^ | 14.96±8.05^b^ |
|  | **Pg_S3293.6** | 26.10±4.27 | 0 | 0 | 0 |
|  | **Count** | 2 | 1 | 1 | 1 |
| **PPTS** | **Pg_S1770.12** | 35.93±28.31^b^ | 97.86±20.45^a^ | 42.39±20.17^ab^ | 49.81±5.51^ab^ |
|  | **Pg_S0325.7** | 0 | 0 | 0 | 0 |
|  | **Count** | 1 | 1 | 1 | 1 |

Different small letters indicate significant differences in the transcript expression level of the four *Panax* species (*p* < 0.05; one-way ANOVA followed by Duncan’s LSR test).

**Supplementary Table S4. Putative identification of ginsenosides analyzed by LC–MS**; PG, *P. ginseng*; PQ, *P. quinquefolius*; PN, *P. notoginseng*; PV, *P. vietnamensis*; PPD, protopanaxadiol; PPT, protopanaxatriol.

| **ginsenoside type** | **no.** | **identification** | **t_R_ (min)** | **Adduct type** | **Calculated *m/z*** | **observed *m/z*** | **Molecular formula (neutral form)** | **Major fragment ions (m/z)** |
| --- | --- | --- | --- | --- | --- | --- | --- | --- |
| PPT | **1** | notoG-R1 isomer | 5.17 | [M−H]^−^ | 931.5266 | 931.5256 | C_47_H_80_O_18_ | 799 [M−Xyl−H]^−^  637 [M−Xyl−Glc−H]^−^  475 [M−Xyl−2Glc−H]^−^ |
|  | **2** | notoG-R1 | 5.69 | [M−H]^−^ | 931.5266 | 931.5277 | C_47_H_80_O_18_ | 799 [M−Xyl−H]^−^  637 [M−Xyl−Glc−H]^−^  475 [M−Xyl−2Glc−H]^−^ |
|  | **3** | Floral-G-M/N | 5.80 | [M−H]^−^ | 1077.5846 | 1077.5841 | C_53_H_90_O_22_ | 945 [M−Ara−H]^−^  931 [M−Rha−H]^−^  799 [M−Rha−Ara−H]^−^  783 [M−Ara−Glc−H]^−^  637 [M−Rha−Ara−Glc−H]^−^  475 [M−Rha−Ara−2Glc−H]^−^ |
|  | **4** | G-Rg1 | 8.16 | [M+FA−H]^−^ | 845.4899 | 845.4915 | C_42_H_72_O_14_ | 637 [M−Glc−H]^−^  475 [M−2Glc−H]^−^ |
|  | **5** | G-Re | 8.59 | [M−H]^−^ | 945.5423 | 945.5446 | C_48_H_82_O_18_ | 783 [M−Glc−H]^−^  637 [M−Glc−Rha−H]^−^  475 [M−2Glc−Rha −H]^−^ |
|  | **7** | 6'-O-acetyl-G-Rg1 | 11.69 | [M−H]^−^ | 841.4950 | 841.4972 | C_44_H_74_O_15_ | 799 [M−Ac−H]^−^  637 [M−Ac−Glc−H]^−^  475 [M−Ac−2Glc−H]^−^ |
|  | **8** | m-G-Re | 13.06 | [M−H]^−^ | 987.5529 | 987.5543 | C_50_H_84_O_19_ | 945 [M−Ac−H]^−^  783 [M−Ac−Glc−H]^−^  637 [M−Ac−Glc−Rha−H]^−^  475 [M−Ac−2Glc−Rha −H]^−^ |
|  | **9** | 6'-O-acetyl-G-Rg1 isomer | 13.90 | [M+FA−H]^−^ | 887.5004 | 887.5017 | C_44_H_74_O_15_ | 799 [M−Ac−H]^−^  637 [M−Ac−Glc−H]^−^  475 [M−Ac−2Glc−H]^−^ |
|  | **10** | 6'''-O-Acetyl-G-Re | 14.31 | [M−H]^−^ | 987.5529 | 987.5546 | C_50_H_84_O_19_ | 945 [M−Ac−H]^−^  783 [M−Ac−Glc−H]^−^  637 [M−Ac−Glc−Rha−H]^−^  475 [M−Ac−2Glc−Rha −H]^−^ |
|  | **15** | G-F3 | 16.87 | [M−H]^−^ | 769.4738 | 769.4755 | C_41_H_70_O_13_ | 637 [M−Glc −H]^−^  475 [M−Ara−Glc −H]^−^ |
|  | **16** | notoG-Rt | 17.08 | [M−H]^−^ | 841.4950 | 841.4944 | C_44_H_74_O_15_ | 799 [M−Ac−H]^−^  475 [M−Ac−2Glc−H]^−^ |
|  | **18** | G-Rh1 | 18.16 | [M+FA−H]^−^ | 683.4370 | 683.4401 | C_36_H_62_O_9_ | 475 [M−Glc−H]^−^ |
|  | **19** | G-Rg2 | 18.24 | [M−H]^−^ | 783.4895 | 783.4904 | C_42_H_72_O_13_ | 637 [M−Rha−H]^−^  475 [M−Rha−Glc −H]^−^ |
|  | **22** | 6'-O-Acetyl-G-Rh1 | 19.89 | [M+FA−H]^−^ | 725.4476 | 725.4467 | C_38_H_64_O_10_ | 637 [M−Ac−H]^−^  475 [M−Ac−Glc−H]^−^ |
|  | **39** | 6'-O-Acetyl-G-Rg2 | 24.43 | [M−H]^−^ | 825.5000 | 825.5009 | C_44_H_74_O_14_ | 783 [M−Ac−Rha−H]^−^  637 [M−Ac−Rha−H]^−^  475 [M−Ac−Rha−Glc −H]^−^ |
| PPD | **17** | G-Ra3 | 17.30 | [M−H]^−^ | 1239.6374 | 1239.6388 | C_59_H_100_O_27_ | 1107 [M−Ara−H]^−^  945 [M−Ara−Glc−H]^−^  783 [M−Ara−2Glc−H]^−^ |
|  | **20** | G-Rb1 | 18.90 | [M−H]^−^ | 1107.5951 | 1107.5979 | C_54_H_92_O_23_ | 945 [M−Glc−H]^−^  783 [M−2Glc−H]^−^  621 [M−3Glc−H]^−^  459 [M−4Glc−H]^−^ |
|  | **21** | m-G-Rb1 | 19.76 | [M−CO_2_−H]^−^ | 1149.6057 | 1149.6065 | C_57_H_94_O_26_ | 1107 [M−mal−H]^−^  1089 [M−mal−H_2_O−H]^−^  945 [M−mal−Glc−H]^−^  783 [M−mal−2Glc−H]^−^ |
|  | **23** | G-Rc | 20.24 | [M−H]^−^ | 1077.5846 | 1077.5848 | C_53_H_90_O_22_ | 945 [M−Ara−Glc−H]^−^  783 [M−Ara−Glc−H]^−^  621 [M−Ara−2Glc−H]^−^  459 [M−Ara−3Glc−H]^−^ |
|  | **25** | m-G-Rb1 isomer1 | 20.71 | [M−CO_2_−H]^−^ | 1149.6057 | 1149.6093 | C_57_H_94_O_26_ | 1107 [M−mal−H]^−^  1089 [M−mal−H_2_O−H]^−^  945 [M−mal−Glc−H]^−^  783 [M−mal−2Glc−H]^−^ |
|  | **26** | m-G-Rc | 21.16 | [M−CO_2_−H]^−^ | 1119.5951 | 1119.5964 | C_55_H_91_O_23_ | 1077 [M−mal−H]^−^  945 [M−mal−Ara−Glc−H]^−^  783 [M−mal−Ara−Glc−H]^−^  621 [M−mal−Ara−2Glc−H]^−^  459 [M−mal−Ara−3Glc−H]^−^ |
|  | **28** | m-G-Rb1 isomer2 | 21.78 | [M−CO_2_−H]^−^ | 1149.6057 | 1149.6078 | C_57_H_94_O_26_ | 1107 [M−mal−H]^−^  1089 [M−mal−H_2_O−H]^−^  945 [M−mal−Glc−H]^−^  783 [M−mal−2Glc−H]^−^ |
|  | **29** | G-Rb2 | 21.84 | [M−H]^−^ | 1077.5846 | 1077.5884 | C_53_H_90_O_22_ | 945 [M−Ara−H]^−^  783 [M−Ara−Glc−H]^−^  621 [M−Ara−2Glc−H]^−^  459 [M−Ara−3Glc−H]^−^ |
|  | **30** | G-Rb3 | 22.38 | [M−H]^−^ | 1077.5846 | 1077.5842 | C_53_H_90_O_22_ | 945 [M−Xyl−H]^−^  783 [M−Xyl−Glc−H]^−^  621 [M−Xyl−2Glc−H]^−^  459 [M−Xyl−3Glc−H]^−^ |
|  | **32** | m-G-Rb2 | 22.65 | [M−CO_2_−H]^−^ | 1119.5951 | 1119.5950 | C_56_H_92_O_25_ | 1077 [M−mal−H]^−^  945 [M−mal−Ara−H]^−^  783 [M−mal−Ara−Glc−H]^−^  621 [M−mal−Ara−2Glc−H]^−^  459 [M−mal−Ara−3Glc−H]^−^ |
|  | **32** | m-G-Rb3 | 23.02 | [M−CO_2_−H]^−^ | 1119.5951 | 1119.5978 | C_56_H_92_O_25_ | 1077 [M−mal−H]^−^  945 [M−mal−Xyl−H]^−^  783 [M−mal−Xyl−Glc−H]^−^  621 [M−mal−Xyl−2Glc−H]^−^  459 [M−mal−Xyl−3Glc−H]^−^ |
|  | **35** | m-G-Rb2 isomer1 | 23.72 | [M−CO_2_−H]^−^ | 1119.5951 | 1119.5974 | C_56_H_92_O_25_ | 1077 [M−mal−H]^−^  945 [M−mal−Ara−H]^−^  783 [M−mal−Ara−Glc−H]^−^  621 [M−mal−Ara−2Glc−H]^−^  459 [M−mal−Ara−3Glc−H]^−^ |
|  | **36** | m-G-Rb2 isomer2 | 23.93 | [M−CO_2_−H]^−^ | 1119.5951 | 1119.5983 | C_56_H_92_O_25_ | 1077 [M−mal−H]^−^  945 [M−mal−Ara−H]^−^  783 [M−mal−Ara−Glc−H]^−^  621 [M−mal−Ara−2Glc−H]^−^  459 [M−mal−Ara−3Glc−H]^−^ |
|  | **37** | G-Rd | 24.04 | [M−H]^−^ | 945.5423 | 945.5409 | C_48_H_82_O_18_ | 783 [M−Glc−H]^−^  621 [M−2Glc−H]^−^  459 [M−3Glc−H]^−^ |
|  | **38** | m-G-Rb2 isomer3 | 24.14 | [M−CO_2_−H]^−^ | 1119.5951 | 1119.5974 | C_56_H_92_O_25_ | 1077 [M−mal−H]^−^  945 [M−mal−Ara−H]^−^  783 [M−mal−Ara−Glc−H]^−^  621 [M−mal−Ara−2Glc−H]^−^  459 [M−mal−Ara−3Glc−H]^−^ |
|  | **40** | m-G-Rd | 24.45 | [M−CO_2_−H]^−^ | 987.5529 | 987.5527 | C_51_H_84_O_21_ | 783 [M−mal−Glc−H]^−^  621 [M−mal−2Glc−H]^−^  459 [M−mal−3Glc−H]^−^ |
|  | **41** | Gypenoside XVII | 25.25 | [M−H]^−^ | 945.5423 | 945.5431 | C_48_H_82_O_18_ | 783 [M−Glc−H]^−^  621 [M−2Glc−H]^−^  459 [M−3Glc−H]^−^ |
|  | **42** | Acetyl-G-Rd | 25.34 | [M−H]^−^ | 987.5529 | 987.5513 | C_50_H_84_O_19_ | 945 [M−Ac−Glc−H]^−^  783 [M−Ac−Glc−H]^−^  621 [M−Ac−2Glc−H]^−^  459 [M−Ac−3Glc−H]^−^ |
|  | **43** | m-G-Rd isomer | 25.92 | [M−CO_2_−H]^−^ | 987.5529 | 987.5552 | C_51_H_84_O_21_ | 783 [M−mal−Glc−H]^−^  621 [M−mal−2Glc−H]^−^  459 [M−mal−3Glc−H]^−^ |
|  | **44** | notoG-Fe | 26.01 | [M+FA−H]^−^ | 961.5372 | 961.5402 | C_47_H_80_O_17_ | 783 [M−Ara−H]^−^  621 |
|  | **45** | Gypenoside XI | 26.68 | [M+FA−H]^−^ | 961.5372 | 961.5372 | C_47_H_80_O_17_ | 783 [M−Ara−H]^−^  621 [M −Ara−Glc −H]^−^  459 [M −Ara−2Glc −H]^−^ |
|  | **46** | G-Rg3 | 27.65 | [M+FA−H]^−^ | 829.4950 | 829.4972 | C_42_H_72_O_13_ | 621 [M−Glc−H]^−^  459 [M−2Glc −H]^−^ |
| Ocotillol | **6** | pseudoG-F11 isomer | 11.00 | [M−H]^−^ | 799.4844 | 799.4840 | C_42_H_72_O_14_ | 653 [M−Rha−H]^−^  491 [M−Rha−Glc−H]^−^ |
|  | **11** | vinaG-R1 isomer | 14.63 | [M−H]^−^ | 841.4950 | 841.4955 | C_44_H_74_O_15_ | 799 [M−Ac−H]^−^  653 [M−Ac−Rha−H]^−^  491 [M−Ac−Rha−Glc−H]^−^ |
|  | **12** | vinaG-R1 | 15.18 | [M−H]^−^ | 841.4950 | 841.4940 | C_44_H_74_O_15_ | 799 [M−Ac−H]^−^  653 [M−Ac−Rha−H]^−^  491 [M−Ac−Rha−Glc−H]^−^ |
|  | **13** | PseudoG-RT5 | 15.94 | [M+FA−H]^−^ | 699.4320 | 699.4325 | C_36_H_62_O_10_ | 491 [M−Glc−H]^−^ |
| Oleanane | **24** | G-Ro | 20.53 | [M−H]^−^ | 955.4903 | 955.4908 | C_48_H_76_O_19_ | 793 [M−Glc−H]^−^  613 [M−2Glc−H]^−^  455 [M−2Glc−GlcA−H]^−^ |
|  | **27** | G-Ro isomer | 21.30 | [M−H]^−^ | 955.4903 | 955.4923 | C_48_H_76_O_19_ | 793 [M−Glc−H]^−^  613 [M−2Glc−H]^−^  455 [M−2Glc−GlcA−H]^−^ |
|  | **31** | Chikusetsusaponin IV | 22.56 | [M−H]^−^ | 925.4797 | 925.4802 | C_47_H_74_O_18_ | 793 [M−Ara−H]^−^  613 [M−2Glc−H]^−^ |
|  | **34** | zingibroside R1 | 23.61 | [M−H]^−^ | 793.4374 | 793.4381 | C_42_H_66_O_14_ | 613 [M−Glc−H_2_O−H]^−^  455 [M−2Glc−GlcA−H]^−^ |
|  | **47** | Chikusetsusaponin Iva | 27.82 | [M−H]^−^ | 793.4374 | 793.4388 | C_42_H_66_O_14_ | 613 [M−Glc−H_2_O−H]^−^  455 [M−2Glc−GlcA−H]^−^ |
|  | **48** | Chikusetsusaponin IVa isomer | 28.05 | [M−H]^−^ | 793.4374 | 793.4395 | C_42_H_66_O_14_ | 613 [M−Glc−H_2_O−H]^−^  455 [M−Glc−GlcA−H]^−^ |
| etc | **14**^a^ | G-Rf (PG) | 16.00 | [M−H]^−^ | 799.4844 | 799.4857 | C_42_H_72_O_14_ | 637 [M−Glc−H_2_O−H]^−^  475 [M−2Glc−H]^−^ |
|  |  | pseudoG-F11 (PQ) |  |  |  |  |  | 653 [M−Rha−H]^−^  491 [M−Rha−Glc−H]^−^ |

^a^ Peak **14** shows same retention time and *m/z* values in PG and PQ, but different MS/MS fragment ions are observed.**Supplementary Table S5.** **Relative abundances of ginsenosides in the adventitious roots of four *Panax* species (PG, PQ, PN and PV) analyzed by LC–MS**; PG, *P. ginseng*; PQ, *P. quinquefolius*; PN, *P. notoginseng*; PV, *P. vietnamensis*; PPD, protopanaxadiol; PPT, protopanaxatriol.

| **Ginsenoside Type** | **ID No.** | **Metabolite name** | **PG** | **PQ** | **PN** | **PV** |
| --- | --- | --- | --- | --- | --- | --- |
| PPT | **1** | notoG-R1 isomer | 6904.67 ±2627.42 b | 79603.33 ±28539.65 a | 13888.67 ±5120.44 b | 4262.67 ±225.02b |
|  | **2** | notoG-R1 | 1777.33 ±462.29 b | 2188.00 ±790.03 b | 87214.00 ±28543.40 a | 302.00 ±214.00b |
|  | **3** | Floral-G-M/N | 6253.33 ±3630.93 b | 6441.00 ±3294.31 b | 3446.67 ±1236.53 b | 30281.67 ±6091.89a |
|  | **4** | G-Rg1 | 246707.33 ±96693.41 b | 641753.00 ±170286.47 a | 454883.00 ±92951.36 a | 14951.67 ±7225.41c |
|  | **5** | G-Re | 529533.00 ±158994.02 b | 1594391.33 ±352720.02 a | 327974.33 ±36446.20 bc | 63657.33 ±5737.95c |
|  | **7** | 6'-O-acetyl-G-Rg1 | 55485.00 ±25721.63 a | 33584.67 ±1246.48 ab | 61248.33 ±24312.14 a | 724.67 ±186.79b |
|  | **8** | m-G-Re | 50433.33 ±23937.49 a | 9905.00 ±2040.90 b | 1787.33 ±79.13 b | 195.00 ±117.69b |
|  | **9** | 6'-O-acetyl-G-Rg1 isomer | 314.67 ±51.81 b | 81411.33 ±11115.79 a | 357.67 ±296.66 b | 44.67 ±19.73b |
|  | **10** | 6'''-O-acetyl-G-Re | 74.67 ±53.95 c | 18984.33 ±2248.75 b | 60.67 ±24.11 c | 38693.33 ±973.24a |
|  | **15** | G-F3 | 14014.33 ±6008.16 a | 84.00 ±70.89 b | 23124.33 ±9333.71 a | 239.00 ±103.01b |
|  | **16** | notoG-Rt | 21528.00 ±13633.92 a | 39.67 ±8.62 b | 272.33 ±212.05 b | 125.00 ±40.29b |
|  | **18** | G-Rh1 | 9353.00 ±5578.99 b | 25794.67 ±13724.51 a | 9524.33 ±4662.53 b | 3652.00 ±1523.91b |
|  | **19** | G-Rg2 | 178473.67 ±83680.29 a | 67928.33 ±22403.07 b | 97553.00 ±28666.38 ab | 12478.33 ±3843.90b |
|  | **22** | 6'-O-acetyl-G-Rh1 | 18369.00 ±13196.87 | 14468.67 ±3645.89 | 21870.33 ±8712.12 | 522.67 ±253.44 |
|  | **39** | 6'-O-acetyl-G-Rg2 | 77.00 ±15.39 b | 785.67 ±519.15 b | 171.33 ±81.00 b | 26077.33 ±3842.04a |
| PPD | **17** | G-Ra3 | 0.00 ±0.00 b | 3.67 ±6.35 b | 15689.67 ±4047.18 a | 784.67 ±185.79b |
|  | **20** | G-Rb1 | 256452.33 ±82967.10 b | 432307.00 ±119326.59 a | 129357.33 ±43288.09 bc | 12642.67 ±1581.74c |
|  | **21** | m-G-Rb1 | 396878.33 ±120449.36 a | 119157.67 ±31248.90 b | 39999.00 ±16857.68 b | 3442.33 ±608.11b |
|  | **23** | G-Rc | 159010.67 ±50490.17 a | 125989.33 ±19046.69 a | 24874.33 ±8810.42 b | 12373.33 ±4187.45b |
|  | **25** | m-G-Rb1 isomer1 | 36456.67 ±9475.22 b | 47320.00 ±5868.87 a | 9486.00 ±1407.57 c | 969.67 ±205.16c |
|  | **26** | m-G-Rc | 235013.00 ±73419.68 a | 38413.33 ±1723.50 b | 5186.67 ±1772.65 b | 4204.67 ±54.72b |
|  | **28** | m-G-Rb1 isomer2 | 75463.33 ±24972.05 a | 18735.67 ±5112.32 b | 5694.33 ±2364.60 b | 470.67 ±183.94b |
|  | **29** | G-Rb2 | 179505.33 ±57508.93 a | 160394.00 ±58344.25 a | 8806.33 ±1655.81 b | 9230.00 ±2697.92b |
|  | **30** | G-Rb3 | 29112.67 ±9771.21 b | 412496.00 ±157234.36 a | 75855.67 ±18391.17 b | 128736.00 ±46006.93b |
|  | **32** | m-G-Rb2 | 280546.67 ±87744.35 a | 48512.00 ±13730.23 b | 1749.33 ±541.04 b | 2513.67 ±170.77b |
|  | **32** | m-G-Rb3 | 93646.00 ±31196.44 a | 131045.00 ±40755.94 a | 26473.00 ±5958.05 b | 40106.00 ±1251.25b |
|  | **35** | m-G-Rb2 isomer1 | 3201.33 ±1168.71 c | 11360.33 ±2802.04 bc | 34451.00 ±10109.29 a | 18735.00 ±4302.32b |
|  | **36** | m-G-Rb2 isomer2 | 55348.33 ±19192.34 a | 8671.33 ±2890.87 b | 961.67 ±325.91 b | 1073.00 ±221.78b |
|  | **37** | G-Rd | 75775.00 ±23813.24 a | 63982.00 ±19279.40 a | 17704.33 ±6189.14 b | 50979.33 ±21753.52ab |
|  | **38** | m-G-Rb2 isomer3 | 8042.00 ±2237.54 b | 22217.67 ±7299.26 a | 3779.00 ±1357.89 b | 6613.67 ±461.59b |
|  | **40** | m-G-Rd | 173680.33 ±77545.85 a | 38160.67 ±6862.06 b | 9593.67 ±3269.69 b | 18932.67 ±4575.25b |
|  | **41** | Gypenoside XVII | 49036.00 ±22270.64 ab | 64590.00 ±37864.76 a | 4960.33 ±1037.42 c | 9064.67 ±2620.85bc |
|  | **42** | Acetyl-G-Rd | 32438.67 ±15260.80 a | 7745.00 ±1506.66 b | 1862.00 ±677.45 b | 3017.33 ±602.65b |
|  | **43** | m-G-Rd isomer | 40343.67 ±13616.45 a | 26426.00 ±11501.74 a | 1964.67 ±490.95 b | 6247.00 ±824.99b |
|  | **44** | notoG-Fe | 27031.33 ±10919.99 a | 13444.33 ±6046.50 b | 7050.33 ±1874.29 b | 12786.33 ±1461.93b |
|  | **45** | Gypenoside Xi | 18602.00 ±7410.93 b | 15997.67 ±3894.22 b | 13847.00 ±4115.66 b | 85632.33 ±12692.46a |
|  | **46** | G-Rg3 | 11416.33 ±3819.38 b | 7251.00 ±3773.33 bc | 1204.33 ±199.14 c | 23983.33 ±6256.88a |
| Ocotillol | **6** | pseudoG-F11 isomer | 673.67 ±539.87 c | 46246.33 ±13087.93 b | 696.00 ±310.35 c | 171868.67 ±33176.65a |
|  | **11** | vinaG-R1 isomer | 46.33 ±34.79 b | 377.00 ±109.09 b | 55.33 ±36.36 b | 95302.33 ±6439.70a |
|  | **12** | vinaG-R1 | 46.67 ±34.85 b | 4546.67 ±796.21 b | 54.33 ±26.63 b | 702381.33 ±55755.08a |
|  | **13** | PseudoG-RT5 | 33.33 ±16.50 b | 65616.00 ±24700.52 a | 170.33 ±81.19 b | 62.67 ±45.61b |
| Oleanane | **24** | G-Ro | 220338.00 ±78466.01 a | 38219.00 ±3672.83 b | 328.00 ±221.47 b | 2531.67 ±1407.40b |
|  | **27** | G-Ro isomer | 36710.33 ±15259.90 ab | 86042.67 ±54032.62 a | 5713.00 ±680.51 b | 287.67 ±136.57b |
|  | **31** | Chikusetsusaponin IV | 15331.67 ±3847.29 b | 25186.33 ±4666.31 a | 419.67 ±308.35 c | 83.67 ±24.42c |
|  | **34** | zingibroside R1 | 195744.00 ±103022.12 a | 28507.00 ±2428.79 b | 135.00 ±127.18 b | 291.33 ±57.29b |
|  | **47** | Chikusetsusaponin Iva | 108076.33 ±39541.15 a | 23924.67 ±7074.17 b | 984.33 ±296.33 b | 24411.67 ±6335.25b |
|  | **48** | Chikusetsusaponin IVa isomer | 18821.33 ±9344.42 b | 69729.33 ±37558.15 a | 24322.00 ±2506.77 b | 14942.00 ±2742.92b |
| etc | **14** | G-Rf (RG) / pseudoG-F11 (PQ) | 142620.33 ±58488.37 b | 1079403.33 ±220120.34 a | 1005.67 ±393.65 b | 591.67 ±971.20b |

Different small letters indicate significant differences in the accumulated compounds of the four *Panax* species (*p* < 0.05; one-way ANOVA followed by Duncan’s LSR test).


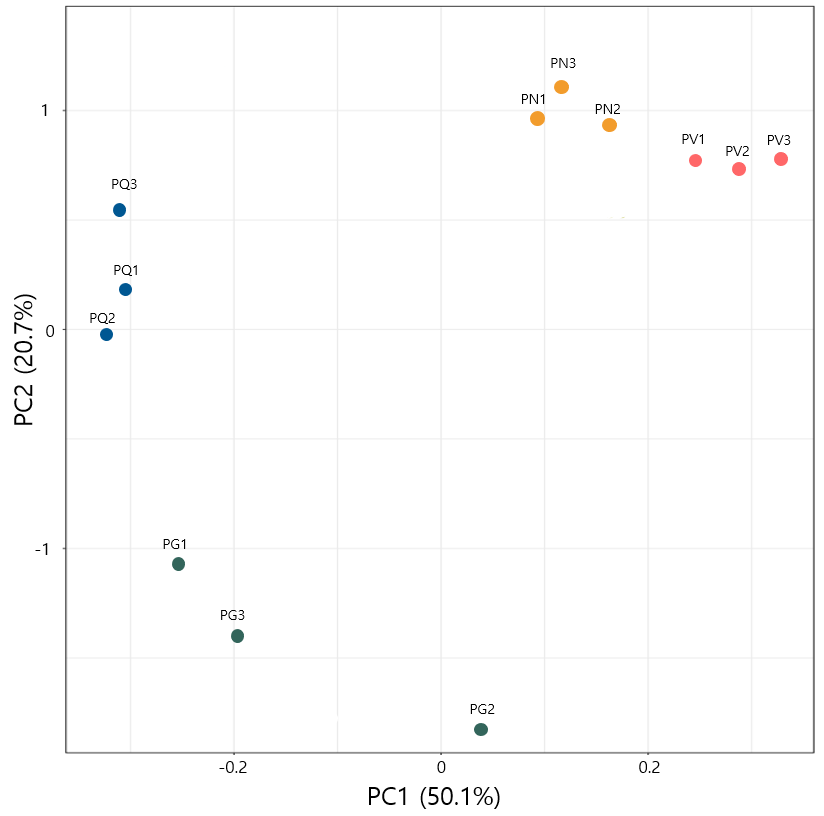


**Supplementary Fig. S1. The principal component analysis (PCA) score plot of the expression data of adventitious roots from four *Panax* species (PG, PQ, PN and PV).** PG, *Panax ginseng*; PQ, *Panax quinquefolius*; PN, *Panax notoginseng*; PV, *Panax vietnamensis***.**


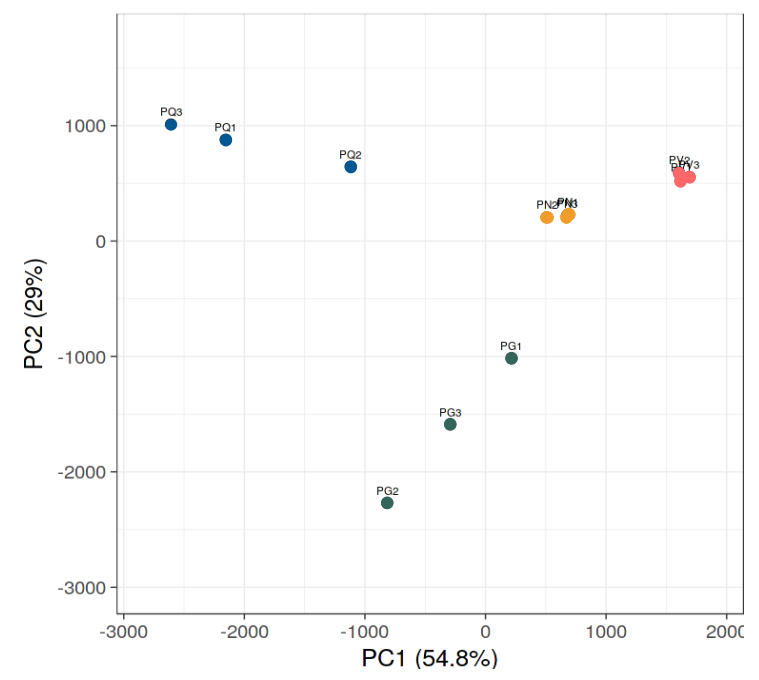


**Supplementary Fig. S2. The principal component analysis (PCA) score plot of relative ginsenoside abundance of adventitious roots from four *Panax* species (PG, PQ, PN and PV).** Pareto scaling was applied and SVD with imputation was used to calculate PCs. PG, *Panax ginseng*; PQ, *Panax quinquefolius*; PN, *Panax notoginseng*; PV, *Panax vietnamensis***.**
